# An Expert Chemist

**Published:** 1888-10

**Authors:** 


### *AN EXPERT CHEMIST.*

Cases are so numerous now-a-days in which the services of an expert chemist are required, that we desire to call the attention of the professional public to the fact that it can be most skillfully and conscientiously served at the hands of John A. Miller, Ph. D., who has just returned to this city from abroad, and who has especially fitted himself for this work. Dr. Miller graduated in 1885 from the University of Illinois, receiving the degree of B. S., and to which the University added the degree of Master of Sciences last June. After his graduation from the University of Illinois, three years ago, he went to Germany, where he entered upon his chemical studies at the University of Berlin. Here he was under such celebrated teachers as A. H. von Hofman, Rammelsberg, Fresenius, von Helmholtz, and many others, and graduated from the University a few weeks ago with high honors, receiving the degrees of M. A. and Ph. D. He also has been honored by being made a member of the Berlin Chemical Society, and a Fellow of the London Chemical Society.

The training that Dr. Miller has thus received eminently fits him for the duties of an expert chemist. In preparation and ability he has no equal in this part of the country, and cases ot poisoning, special chemical analysis, etc., can be entrusted to him with the utmost confidence. He succeeds the late Dr. Davidson in the Medical Department of Niagara University in medical chemistry and toxicology, and has opened a laboratory in the College building. Physicians, coroners, and others, can submit analytical work to him with the assurance that it will be most carefully and thoroughly done.

